# Comparison of the Biological Behavior and Topographical Surface Assessment of a Minimally Invasive Dental Implant and a Standard Implant: An In Vitro Study

**DOI:** 10.3390/ma15217540

**Published:** 2022-10-27

**Authors:** Nina Attik, Marina Phantarasmy, Hazem Abouelleil, Charlène Chevalier, Aurore Barraco, Brigitte Grosgogeat, Arnaud Lafon

**Affiliations:** 1 Laboratoire des Multimatériaux et Interfaces, UMR CNRS 5615, Université Claude Bernard Lyon 1, Université de Lyon, 69622 Villeurbanne, France; 2 Faculté d’Odontologie, Université Claude Bernard Lyon 1, Université de Lyon, 69372 Lyon, France; 3 Hospices Civils de Lyon, Service d’Odontologie, 69003 Lyon, France

**Keywords:** biomaterials, minimally invasive dental implant, cytocompatibility, mineralization, human gingival fibroblasts, osteoblasts, topographical properties

## Abstract

The current study aimed to assess the topographical and physical properties of a minimally invasive implant (MagiCore^®^: MC^®^, InnosBioSurg, IBS) and to evaluate its biological behavior compared to a gold standard implant (NobelParallel™: NB™, Nobel Biocare™). After surface characterization, the biological behavior assessment was conducted regarding human gingival fibroblasts (hGF) and osteoblast-like cells (MG63). Roughness values for NB^TM^ were Ra = 1.28 µm and for MC^®^ they were Ra = 2.02 µm. Alamar Blue^TM^ assay LIVE/DEAD^TM^ staining results indicated equivalent biological development regarding both cell types for the two implants. Significant enhancement was found for hGF ALP activity in the presence of the two tested implants in a time-dependent manner from day 7 to day 14 (** *p* < 0.01). Alizarin red staining demonstrated significant calcium deposition enhancement when cells were interfaced with the NB™ compared to the MC^®^ implant (** *p* < 0.05). Moreover, SEM and confocal imaging revealed good cell adhesion with a denser cellular layer on the MC^®^ than the NB™ surface. The MC^®^ cytocompatibility was ranked as equivalent to the gold standard implant despite the surface properties differences. These findings provide new insights about the minimally invasive implant’s biological behavior and its potential clinical implication in different implantology situations.

## 1. Introduction 

Implant rehabilitation is an ideal solution for unitary or multiple missing teeth. Moraschini et al. describe 94.6% implant survival over a period of 10 years of follow up [1]. Despite a decrease in edentulous patients, an American study suggested that 23% of those patients will have dental implants by the year 2026 [2]. Currently, over two hundred implant brands are identified worldwide [3]. Moreover, new biomaterials and technological innovations allow suppliers to establish new concept treatments including creative design or surface treatments. 

Implants can provide important solutions to dental and orthopedic problems, yet their failure rates in senile or medically compromised patients are still high [4]. In light of this, there is a real need to develop minimally invasive procedures with new implant designs to simplify surgical protocols and increase patient post-surgical comfort. Thus, minimally invasive treatments across flapless surgery and one-piece implants were developed to decrease bleeding disorders and infectious risks. Additionally, it could extend the indications for high-risk medical and terminal bone atrophy patients which is a current public health issue in oral implantology [5] since the scar tissue resulting from surgery could easily be prone to bacterial infections [6]. There are several types of implant failures of which 81.9% are estimated to be due to peri-implantitis [7]. On the other hand, osseointegration amelioration lies in implantation rapidity, which has been widely studied, underlining the need to use a rough surface instead of a turned surface. Along the same lines, rougher implant surfaces were reported to be less retentive of bacterial plaque than turned implant surfaces [8,9,10]. Creating and maintaining healthy and thick peri-implant soft tissue to prevent infections and implant failure is one of the main current research and clinical practice aims [11,12]. Early implant failure could be attributed to design and surface characteristics that affect primary stability [13]. In this context, assessing the effect of implant surface properties using representative cells of bone and soft dental tissues could be a first step in their in vitro evaluation. In vitro, surface properties were reported to influence cell behavior such as the increase in MG63 cell proliferation and attachment with moderate surface roughness [14]. In general, osteoblast adhesion on biomaterials was reported to be related to their surface topography. Moreover, cell spreading and cytoskeleton organization were found to be mainly affected by the surface morphology which could influence many functions [15,16,17].

Various surface treatments including physicochemical and morphological surface modifications have been developed to increase surface wettability and hence osseointegration. Moreover, optimizing surface properties could help to prevent peri-implant diseases [10,18,19]. Currently, sandblasting and surface etching are the most common surface treatments; some use oxidation to create a thick TiO_2_ layer such as Nobel Biocare™’s implant-specific surface. Surface micro geometries induced by surface treatments are characterized by their roughness with two-dimensional parameter Ra: arithmetic roughness average or three-dimensional Sa: arithmetical mean height [20]. According to the literature, titan plasma spray surface modification has the highest Ra (2.1–37.9 µm), the oxidized surface has an intermediary Ra (1.35–2 µm), and sandblasting surface Ra is the lowest (0.9–1.09 µm) [21]. Surfaces are considered moderately rough when Sa is between 1.1–2 µm [22]. Long-term success could not be totally attributed to surface roughness, as the choice of the implant system, the practitioner, or the abutments are also factors of success. Furthermore, many studies have shown no difference in osseointegration of these distinct surface modifications [23,24]. Current bioactive surface studies are made to stimulate osseointegration and to prevent peri-implantitis [25]. Several in vitro and in vivo studies have shown that Ti6Al4V implants are biocompatible and are able to establish osseointegration equivalent to that of commercially pure titanium (cp-Ti); the titanium TiO_2_ passive surface layer forms a resistant film with a high hardness that protects the surface from corrosion and provides a suitable biological interface with bone [26,27]. 

From clinical perspectives, the new implant MagiCore^®^ was designed with a thin machined neck, allowing the implant to be placed without lifting a flap, even for complex anatomical situations involving conditions with thin crestal bone. Consequently, this could extend the indication to bone atrophy patients. Moreover, the fine, rough threads of the MagiCore^®^(Magic Fin Thread) are of clinical interest, being less aggressive to the fine vestibular cortices and thus avoiding bone compression. At the histological and cellular levels, it is important to validate the fibroblast cell adhesion and proliferation on the new implant neck compared to the neck of a standard implant. This soft tissue barrier observed clinically could provide long-term stability and prevent peri-implantitis through bacterial infiltration as compared to conventional implants. It was recently reported that human gingival fibroblasts modulation, the major cell type in oral peri-implant soft connective tissue, is essential for ensuring early and stable transmucosal tissue for an enhanced soft tissue seal that may be less vulnerable to peri-implant disease [28].

In addition, the osteoblast behavior was investigated to predict the cell response at the bone–implant interface. The focus was on the surface assessment as it is possible to be studied in vitro and “the entire implants” were compared, not only the corresponding “material substrates”, to predict as well as possible the clinical situation. To this end, two representative dental cells were used: MG-63 as representative of bone tissue and hGF as representative of soft tissue. These two tissues could be in contact with dental implants after their placement in the oral cavity. 

The aim of the current in vitro study was to assess the physical, topographical, and biological behavior of two different commercially available dental implants (MagiCore^®^ versus NobelParallel™). One hypothesis was suggested: the studied implants would have the same behavior regarding the two studied cell types (human gingival fibroblasts and osteoblasts) despite differences in surface properties. Thus, cell metabolic activity, cell colonization, and remineralization ability were investigated. The physical properties, as well as the surface topography of the MagiCore^®^ implant, were also assessed and compared to the standard implant from Nobel Biocare™. This implant was chosen as a control as it has significant positive feedback and is currently the most widely used dental implant [3].

## 2. Materials and Methods 

### 2.1. Surface Properties Characterization

#### 2.1.1. Studied Implants

Two implant types were investigated: the MagiCore^®^ (MC^®^), a minimally invasive implant from INNOBIOSURG Co.,Ltd. (Daejeon, Korea), and the NobelParallel™ Conical Connection (NB™) from Nobel Biocare™ (Zurich, Switzerland) was used as a control. Fifteen implants of each implant type were used in the current study. Characteristics of surface treatments and implant thread dimensions are summarized in Table 1. The design and the threads morphology of each implant were analyzed using a 3D function VHX-5HM of a digital microscope (Keyence VHX-7000, Osaka, Japan) (Figure S1 of the Supplementary Material). 

#### 2.1.2. SEM-EDX Analysis 

Implant surface analysis was performed by scanning electron microscopy (SEM, FEI-Quanta 250—Thermo Fisher Scientific, Illkirch-Graffenstaden, France) with an acceleration voltage of 10 kV. The combination with energy-dispersive X-ray spectroscopy mode (EDX) was used to investigate the surface composition of the two studied implant surfaces. 

#### 2.1.3. AFM and Profilometer Surface Roughness Measurement

Surface topography and micro-geometry were analyzed by atomic force microscopy (AFM, Nano Observer Scientec Ibérica Spain—CSI, Park Systems France SARL, ORSAY). Arithmetical mean height (Sa) was calculated on five samples of each implant. Measurements have been normalized using Gwyddion software (Version 2.61, D. Necas & P. Klapetek, Brno, Czech Republic).

Thread dimensions and arithmetic roughness average (Ra) were evaluated by an optical profilometer (Altimet Altisurf500, Phénix V2 software, ALTIMET, Thonon-les-Bains, France) with a 110 nm resolution optical probe (STYL CL4) according to the ISO 4287 recommendations.

#### 2.1.4. Wettability Measurement

The wettability of the implant surfaces was evaluated by contact angle measurements using a sessile drop test with 2 µL of distilled water (EasyDrop, Krüss, Germany). Five measurements were established on one sample of each implant. The NB^TM^ implant apex and the MC^®^ implant support were directly used for this measurement. 

### 2.2. Biological Assessment

#### 2.2.1. Cell Culture

Human gingival fibroblasts (hGF) and osteoblasts (MG63, CRL1427, ATCC) were used. The hGF cells were isolated from healthy gingival tissue biopsies of patients during routine orthodontic extractions. The collection of human dental tissue was conducted in compliance with French legislation (informed consent was obtained from the patients at the University of Lyon 1—Hospices Civils de Lyon, France) following local ethical committee approval. Cells were cultured in Dulbecco’s Modified Eagle’s Medium (DMEM) with 10% fetal bovine serum, 5% penicillin/streptomycin, and 0.2% amphotericin B. Cultures were maintained at 37 °C under a humidified atmosphere of 5% CO_2_ in the air. The medium was changed every 2–3 days. After reaching confluence, cells were trypsinized and resuspended in the culture medium and examined routinely under an inverted microscope. The cells were centrifuged at 1200 rpm/min for 5 min and counted with a Scepter handheld automated cell counter (Millipore, Burlington, MA, USA). After the trypsin removal, the remaining cell pellets were resuspended in a fresh medium for subsequent experiments. Then, cells were seeded in 24-microwell plates at a concentration of 10^4^ cells/mL. In this study, cell biological assessment was established for indirect contact (IC) and direct contact (DC). Indirect contact cells’ eluate was evaluated until day 3, then the cells were fixed by 3.7% formaldehyde. Direct contact cells were maintained for 14 days to assess each implant’s cytocompatibility and fixed to indicate cell morphology. 

#### 2.2.2. Cytotoxicity

LIVE/DEAD^TM^ assay (L3224, Thermo Fisher Scientific, France) was achieved on indirect contact cells on day 1 to evaluate cytotoxicity via analyzing membrane integrity. This reagent is composed of two fluorescent dyes: calcein, which stains live cells green, and ethidium homodimer-1, which only penetrates damaged cells. Cells were observed by epifluorescent microscopy (Eclipse e400, NIKON, Tokyo, Japan).

#### 2.2.3. Metabolic Activity

Cell metabolic activity was measured using the Alamar Blue^TM^ assay (DAL1100, Thermo Fisher Scientific, Illkirch-Graffenstaden France) which is colored blue in its oxidized state and becomes pink when reduced to resorufin by metabolically active cells. After 1 day of IC and after 1, 3, 7, and 10 days of contact cells, Alamar Blue^TM^ solution was added directly into the wells at the final concentration of 10% *v*/*v*, and the plates were incubated at 37 °C for 6 h. The amount of resorufin formed was determined by measuring optical density intensity (570  nm/600  nm) using a microplate reader (Infinite^®^ M200 PRO NanoQuant, Tecan, Lyon, France) and is proportional to the number of metabolically active cells. 

#### 2.2.4. Cells Morphology, Colonization, and Adhesion Using Confocal and SEM Imaging

Fluorescence staining was performed to observe cell morphology and colonization. Indirect and direct contact cell morphology was analyzed using LEICA SP5 X confocal laser scanning microscope (CLSM, LEICA, Wetzlar, Germany) and ZEISS 880 laser scanning microscope (LSM, Carl Zeiss, Oberkochen, Germany). Actin microfilaments were stained by Alexa Fluor^TM^ 488 Phalloidin (A12379, Thermo Fisher Scientific, France) at a 1:100 ratio (green fluorescence). Cell nuclei were identified using propidium iodide (P3566, Thermo Fisher Scientific, Illkirch-Graffenstaden, France) at a 1:3000 ratio (red fluorescence).

Cell adhesion was visualized after 14 days of direct contact. Briefly, hGF and MG63 on NB™ or MC^®^ were fixed using 3.7% formaldehyde in PBS for 30 min and rinsed with PBS. The samples were dehydrated using a graded series of ethanol (25%, 35%, 50%, 75%, 90%, 95%, and 100%). Afterwards, samples were coated using 10 nm of copper, and SEM images were captured (FEI Quanta 250, Thermo Fisher Scientific, Illkirch-Graffenstaden, France).

#### 2.2.5. ALP Quantification

Osteoinduction implant potential was assessed with semi-quantification of alkaline phosphatase which is an osteogenesis early marker. After 7 and 14 days of culture, its quantity was estimated in direct contact cells supernatant through ALP kit assay, used according to the manufacturer’s instructions (K412-500, BioVision Incorporated, Waltham, MA, USA). The ALP enzyme present in the samples will convert the *p*-nitrophenyl phosphate (*p*NPP) substrate to an equal amount of colored *p*-nitrophenol (pNP). The optical density of the resultant-colored reaction product within the supernatant was measured at 405 nm. Finally, ALP activity was calculated according to the following equation:

ALP activity = ([*p*NP] (μmol)/ΔT (min) × V (ml) × D (dilution factor),

with ΔT = reaction time and V = initial volume added to each well.

#### 2.2.6. Alizarin Red S

Further, remineralization ability has been performed in direct contact cells using alizarin red S which targets calcium deposits in the extracellular matrix. After 14 days, 40 mM of alizarin red S solution (pH 4.2) was added to the 24-well plates and incubated for 30 min. Then, quantitative calcium analysis (semi-quantification) of mineralized matrix nodules generated from the cells was performed by adding 10% cetylpyridinium chloride solution (Sigma-Aldrich). The optical density values were read at 560 nm, which represented the relative quantity of mineralization nodules. 

### 2.3. Statistical Analysis

Non-parametric analysis and multiple comparisons were achieved using one-way analysis of variance (ANOVA) with a repetition test followed by post hoc tests using the statistical software SPSS^TM^ (V21.0, IBM, Bloomington, IL, USA). A comparison was performed between the two tested implants (MC^®^ vs NB™). For indirect contact, implant extract exposed cells were also compared to control group cells (cells without any treatment). Results were reported as mean standard deviation (±SD) and statistical significances were accepted at *p* < 0.05 and *p* < 0.01.

## 3. Results

### 3.1. Surfaces Properties Characterization

#### 3.1.1. SEM-EDX Analysis

**Figure 1:**
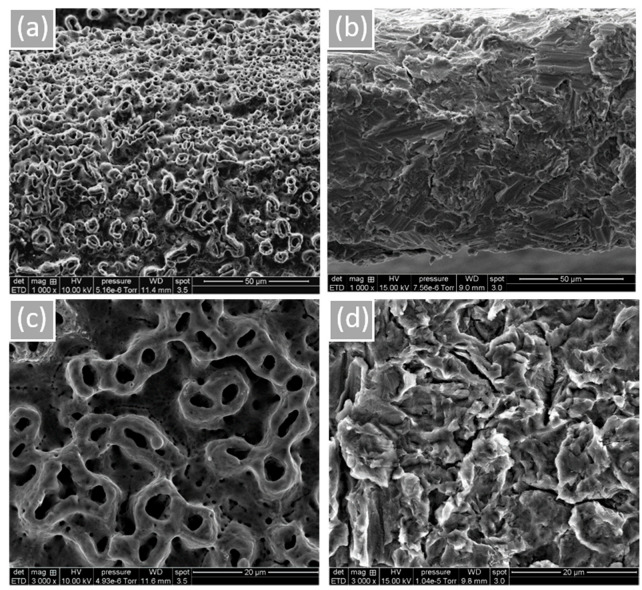
Implant surface morphology observed by SEM. (**a**) NB™, (**b**) MC^®^, (**c**) NB™, and (**d**) MC^®^. Scale bars = 50 µm and 20 µm.

Surface morphology and roughness are shown in Figure 1. Microtubules created by the anodization of NB™ show a rounder appearance. MC^®^’s sandblasted surface is peeled with some prominent peaks. According to the obtained results, more oxygen is noticeable for NB™ (Grade 4) due to the TiO_2_ thick layer formed by electrochemical anodization (based on the technical information). Residual phosphorus from this procedure is also found (Table 2, Figure S2 of the Supplementary Material). Concerning MC^®^ (Grade 5) surface composition, relative alloys aluminum and vanadium were identified.

#### 3.1.2. Surface Roughness 

**Figure 2:**
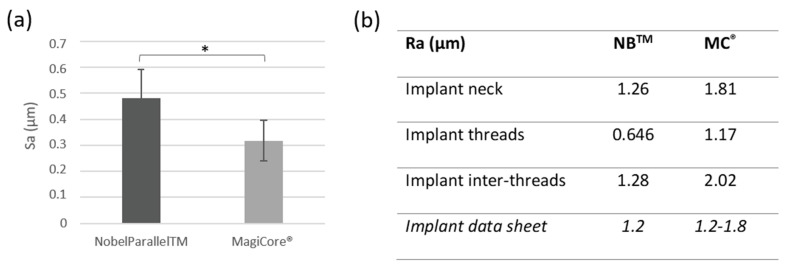
Surface roughness. (**a**) Arithmetical mean height (Sa) (n = 5 ± SD, * *p* < 0.05) and (**b**) Arithmetic roughness average (Ra).

Results are presented in Figure 2. Surface roughness Sa is slightly higher for NB^TM^ (0.48 µm ± 0.11) than MC^®^ (0.31 µm ± 0.07). Both implants’ Ra roughness is considered moderately rough (between 1–2 µm). Surface profilometry roughness Ra is lower for NB™ for the implant inter-threads 1.28 µm vs. 2.02 µm for MC^®^ and 0646 (NB™) vs. 1.17 (MC^®^) for the implant thread Ra values.

#### 3.1.3. Wettability Measurement

**Figure 3:**
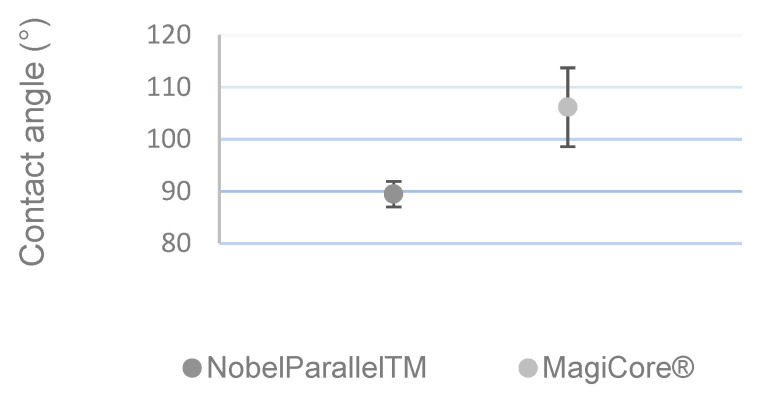
Contact angle measurement (n = 5 ± SD).

Figure 3 shows that MC^®^’s surface (θ = 106.14° ± 7.57) is more hydrophobic than that of NB™ (θ = 89.46° ± 2.45).

### 3.2. Biological Assessment 

#### 3.2.1. Cytotoxicity 

**Figure 4:**
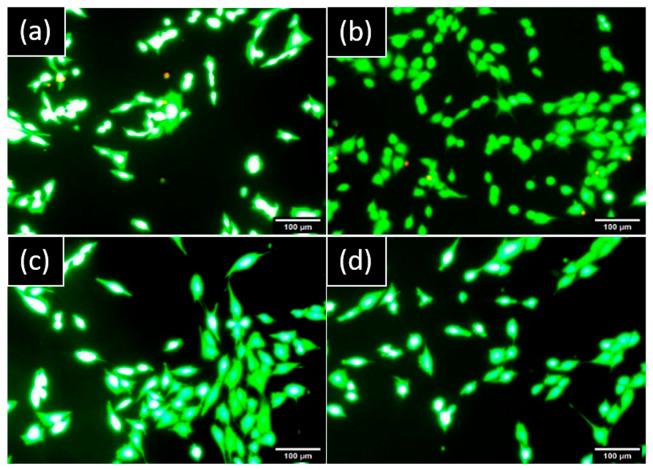
Representative images of cells stained by LIVE/DEAD™ observed by epifluorescence microscopy after one day: hGF in indirect contact with (**a**) NB™ and (**b**) MC^®^. MG63 in indirect contact with (**c**) NB™ and (**d**) MC^®^. Green areas are live cells and red areas are damaged cells. Scale bars = 100 µm.

No cells presented a red marker on hGF and MG63 cultures which demonstrated no damaged cells and a nontoxic component released from both implants (Figure 4). 

#### 3.2.2. Metabolic Activity

**Figure 5:**
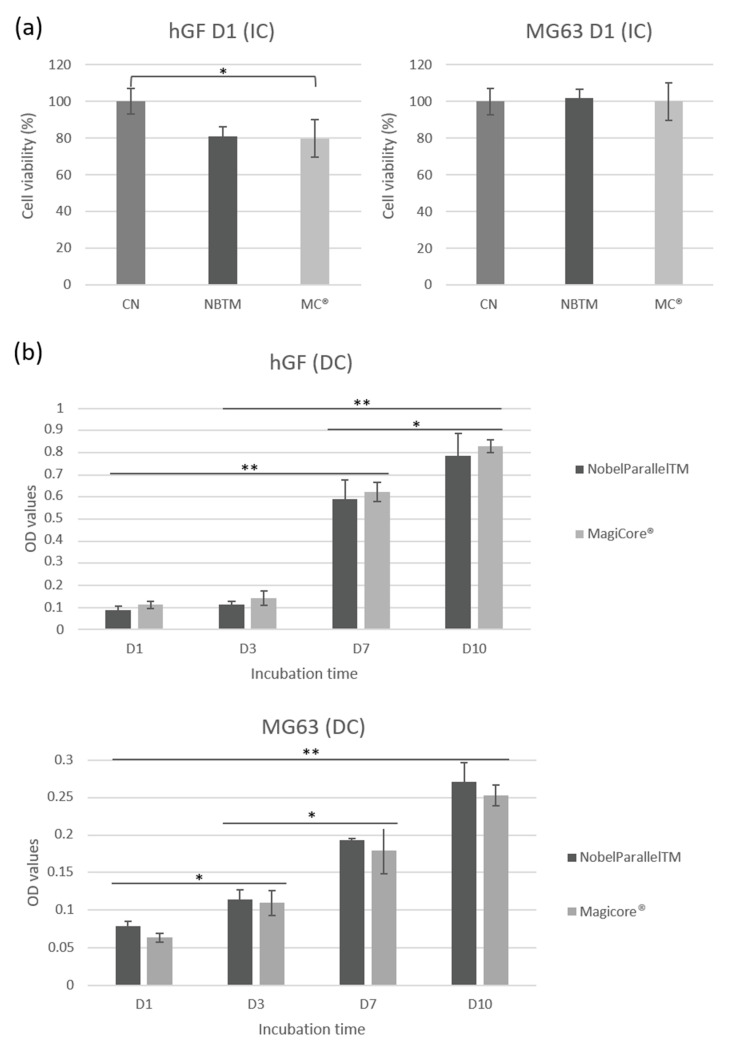
hGF and MG63 cell viability rate. (**a**) Cell metabolic activity is expressed by cell viability (%) for cells exposed to implant extracts. (**b**) Cell metabolic activity is expressed by optical density (OD) values for cells in direct contact with implants (n = 9 ± SD, ** *p* < 0.01 and * *p* < 0.05).

MC^®^ (81%) and NB™ (79.7%) cell viability shows good metabolic activity concerning IC hGF (Figure 5a). Results of MG63 IC show comparably high cell viability for both implants. Concerning DC cells, equivalent hGF metabolic activity is indicated for both surfaces. Lower metabolic activity is detectable for MG63 in direct contact with MC^®^ even though optical density increases during the incubation time (from day 1 to day 10) (Figure 5b).

#### 3.2.3. Cell Morphology, Colonization, and Adhesion Using Confocal and SEM Imaging

Epifluorescence images reveal similar morphology for both hGF and MG63 in indirect contact with NB™ with a cylinder-shaped and round cytoskeleton. Rounder cells are visible with the smallest cytoskeleton in indirect contact with MC^®^ (Figure S3 of the Supplementary Material).

**Figure 6:**
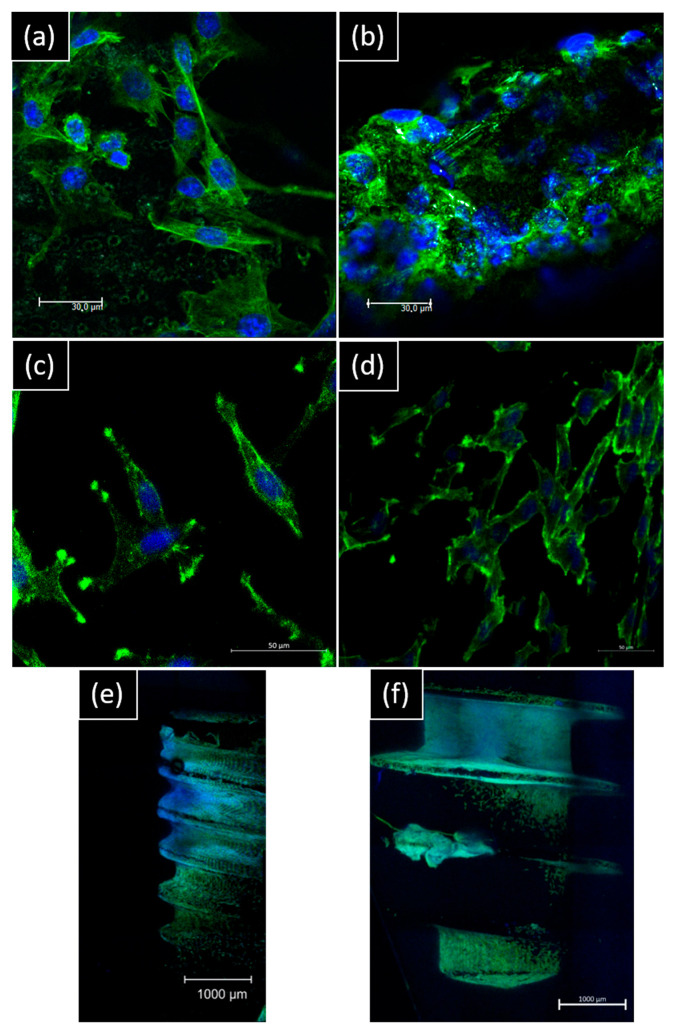
Representative confocal images of hGF and MG63 morphology on day 14 of contact with implants. hGF were in direct contact with (**a**) NB™ and (**b**) MC^®^ (scale bar = 30 µm). MG63 were in direct contact with (**c**) NB™ and (**d**) MC^®^ (scale bar = 50 µm); (**e**) NB™ and (**f**) MC^®^ (scale bar = 1000 µm). Nucleus-blue (DAPI) and cytoskeleton-green (ALEXA Fluor^TM^ 488 Phalloidin).

For direct contact, morphology spreading by confocal imaging is different on the surface: for NB™, longer pseudopods spread over microtubules. For MC^®^, the cell layer is denser, and cells look more adapted to the surface topography (Figure 6). 

**Figure 7:**
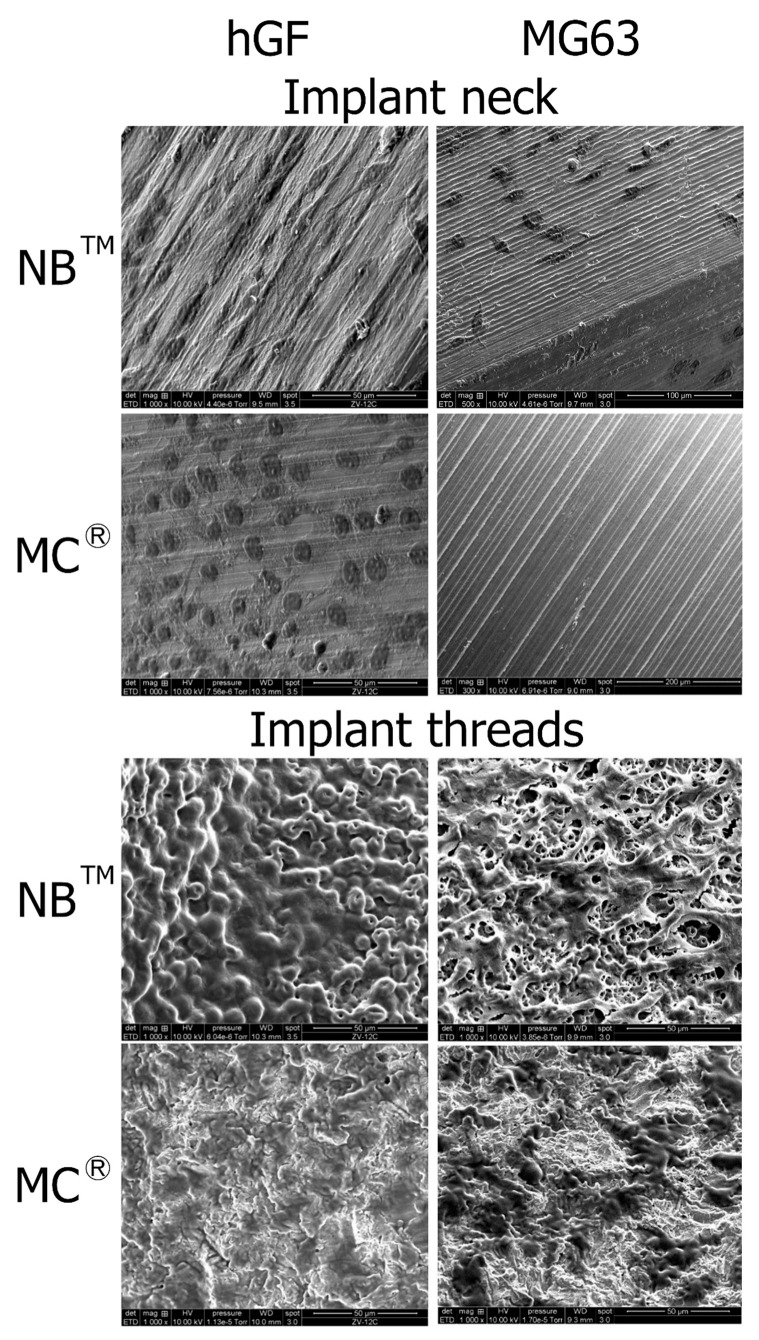
Cell adhesion observed by SEM at the implant necks and implant threads. hGF and MG63 cells were in direct contact with NB™ and MC^®^. Scale bars = 50 µm, 100 µm and 200 µm.

SEM observations show hGF spreading from the post to the implant apex for both implants. On the MagiCore^®^post, more cells are detectable with dense cell layers. The hGF cytoskeleton follows the micro-grooving of the NB™ post. The cell layer recovers microtubules while dark spot cells are more distinguishable on sandblasted surfaces. MG63 osteoblasts are found on the NB™ posts but none on the MagiCore^®^ posts. The cell layer on NB™ with long pseudopods is visible on the threads while elongated cells are found on the MC^®^ threads. Cells adapted differently on the abrasive surface of MC^®^ (Figure 7).

#### 3.2.4. Quantitative Assay of ALP Activity

**Figure 8:**
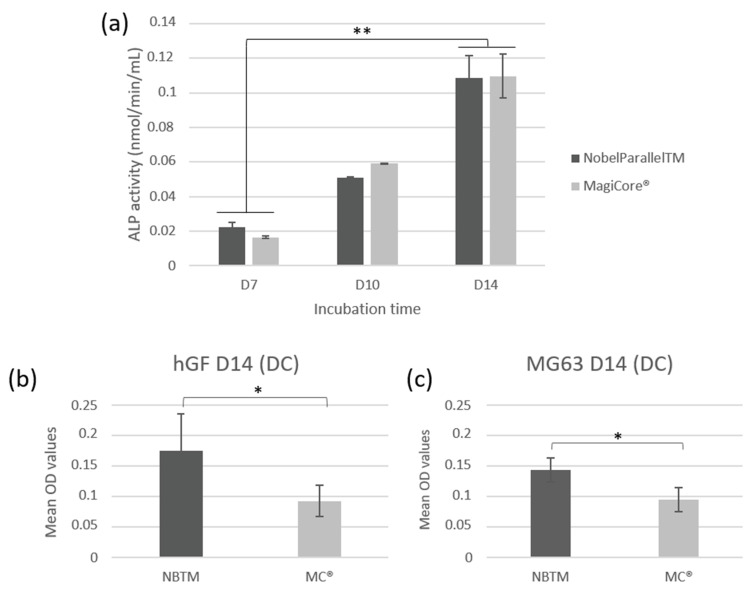
Cellular mineralization: (**a**) extracellular ALP activity of hGF cells following contact with the implants (n = 7 ± SD, ** *p* < 0.01). Quantification of calcium deposition of cells using alizarin red staining on (**b**) hGF and (**c**) MG63 cells (n = 7 ± SD, * *p* < 0.05).

Both tested implants showed significantly increased ALP activity at day 7, day 10, and day 14 (*p* < 0.01). The increase in ALP activity in a time-dependent manner from day 7 to day 14 indicates that hGF cells have developed early mineralization potential (Figure 8a).

#### 3.2.5. Alizarin Red S Staining—Mineralization

Results show higher calcium deposit optical density for NB™ than MC^®^ for both types of cells (Figure 8b,c). However, optical microscopy observation indicates larger remineralization nodules in the extracellular matrix of MC^®^ (Figure S4 of the Supplementary Material).

## 4. Discussion 

Dental implant failures and post-surgical complications remain a public health issue; thus, minimally invasive procedures were developed in order to secure surgical benefits and enhance patient post-surgical comfort through the use of an atraumatic act [29,30]. Preserving a thick keratinized soft gingival tissue is one of the major challenges in order to obtain good healing and create a sufficiently dense epithelial barrier in order to avoid peri-implantitis [11,12]. In different clinical situations, peri-implantitis could be induced by different factors such as implant surface topography, chemical composition, and the release of different harmful elements [31]. Previous studies have shown that released residues from dental implants could cause hypersensitivity of the surrounding tissue [32,33,34,35]. The current results demonstrated that both implants induced no cytotoxic effect in indirect contact and low cytotoxicity in direct contact with both gingival fibroblast and osteoblast cells. It is known that aluminum and vanadium could be released from the implant titanium substrate (grade 5) due to corrosion. It is accepted that a high rate of aluminum could induce bone calcification defects and neurological disorders. Moreover, the toxicity of vanadium could result in a severe disruption of enzymatic reactions with diverse outcomes such as cellular immune deficiencies [36,37]. In agreement with our results, both aluminum and vanadium as part of the Grade 5 composition were reported to be under the toxicity threshold according to the ISO 5832 recommendations [38]. 

Adequate contact between cells and the dental implant surface is very important for the assessment of their biocompatibility in vitro. According to the methods reported in the ISO 10993-5 standard [39], there are two approaches to the evaluation of the in vitro biological assessment of such implants. Some studies have assessed the effects of implants placed in direct contact with cells [40,41,42,43,44], while others have assessed the effects through material extracts collected from the biological medium (indirect contact) [45].

Three endpoints were investigated in the current study to assess the biological behavior of the studied implants: cell viability, cell morphology, and cell mineralization potential ability regarding human gingival fibroblasts (hGF) and human osteoblast-like cells (MG63). Many in vitro studies have used hGF to determine the effect of different surface treatments on cell adhesion and spreading to predict the epithelial attachment potential and soft tissue sealing in the clinical situation [46,47]. Furthermore, the biological response of the implant surface is assessed using osteoblast MG63 cells [41,48] and various osteogenic factors such as alkaline phosphatase expression which is mainly quantified as representative of the studied surface’s mineralization potential [26,42,49]. In agreement with our study, Wagner et al. 2021 investigated both HGF and MG63 cells to compare the effect of cold atmospheric plasma surface modifications on cell behavior using dental implant titanium and zirconia discs [50]. Furthermore, future investigations for an accurate composition analysis using XRD of the two compared implants could provide relevant information and help to understand these implants’ effect on cell behavior in vitro and tissue integration in vivo. 

The present study assessed two distinct implants by their surface treatments (sandblasted vs. oxidation). The treatment based on anodic oxidation was reported to be the best method of surface modification with the ability of bone formation enhancement through the formation of an electrochemically porous amorphous TiO_2_ film and of micropores and nanopore surfaces [51,52,53]. Thus, an oxidized surface was chosen as the control because it has been subjected to numerous investigations and has demonstrated its long-term effectiveness [54,55,56]. On the other hand, sandblasting is one of the most commonly used surface treatments able to generate a symmetrical distribution of peaks and valleys on the treated surfaces [18,20,57].

Studying the behavior of specific cells in contact with implants with various surface topography could provide relevant information and predict their in vivo performance regarding targeted tissues. Cell morphology was dependent on the surface types, while indirect contact cells on glass slides were more elongated for both cell types; cells in direct contact with implants had different cell morphology on NB^TM^ and MC^®^ due to their respective surface treatments. Cells had the ability to adapt their morphology to monitor the surface microstructure of the MC^®^, while on the NB^TM^ surface, cells spread out over the pores (Figure 4). According to Yamagami et al., the oxidized surface showed better osseointegration ability when the roughness Ra is moderate, around 2.7 µm [58]. Furthermore, it has been reported that in oral and maxillofacial surgery, moderately rough implants demonstrated higher longevity than minimally rough implants in the maxillary arch but not in the mandible [20]. Moreover, no evidence could be found in the literature that any particular type of dental implant can be shown to be more clinically successful in the long term. However, rough surfaces were reported to allow higher bone–implant contact values and better bone apposition, including cell proliferation and migration stimulation, compared to smooth surfaces [18,59,60]. 

In the current study, the obtained profilometry data (Figure 2b), which performed according to the ISO 4287 recommendations, confirm that both surfaces are moderately rough with a Ra value between 1 and 2 µm. On the other hand, a previous meta-analysis stated that the risk of crestal bone loss could be related to the increase in surface roughness [22]. Furthermore, in the study by Rabel et al., the authors showed that organization and intrinsic surface topography affect the osteoblasts’ morphogenesis [61]. Cell morphology depended on surface microgeometry which can impact the expression of cellular phenotype [62,63,64]. Micrometer-scale roughness values (Sa and Ra) are commonly used to characterize topographical dental implants, according to surface amplitude, and this effect could reflect behavior at the supra-cellular level [61]. In general, dental implant surface modifications were previously investigated and are still the focus of recent research, being a fundamental feature for successful osseointegration [60,65]. It has been shown in several studies that hydrophobic surfaces are less suitable for cell adhesion and spreading [62,66]. In addition to the currently investigated parameters (cell adhesion, cell spreading, and colonization), the assessment of focal adhesion biomarkers such as intercellular cell adhesion molecule 1 (ICAM-1) expression or vinculin fluorescent staining at the focal adhesion area could provide additional information regarding cell behavior in contact with the tested implant surfaces.

Despite the limitation of this in vitro study (short incubation time, implant geometrical design, in vitro tests are not fully representative of in vivo assays), the cytocompatibility data demonstrated a good biological behavior of the minimally invasive implant equivalent to the gold standard implant, and the initial hypothesis that both implant surfaces have equivalent biological behavior was validated. Both implants demonstrated equivalent results regarding metabolic activity quantification and membrane integrity evaluation even though surface treatments were different. From a clinical perspective, the obtained conclusions could contribute to the use of this minimally invasive implant MC^®^ in different clinical situations. Its flapless placement protocol would extend its indications for patients with bone atrophy or other pathologies that initially contraindicate implant placement. Further investigations are needed to evaluate the effect of accelerated aging on biological behavior and to assess the degradation profile and the possible release of components after an extended period. Moreover, the current in vitro-identified effects on cell adhesion, colonization, and mineralization ability need to be confirmed *in vivo*. To this aim, an in vivo investigation is currently being conducted by our group to assess and compare the studied implants in terms of tissue integration, and to determine to what extent these in vitro results have effects within the complexity of a biological animal system. Finally, correlations between current in vitro data and future histological data could provide a complete analysis of the bone and soft tissue interface outcomes.

## 5. Conclusions 

The current in vitro assessment allowed us to rank the biocompatibility of the minimally invasive implant MagiCore^®^ as equivalent to the gold standard implant NobelParallel™. This favorable biological behavior was demonstrated regarding human gingival fibroblasts and human osteoblast-like cells: 

Enhanced cytocompatibility and low cytotoxicity;

Improved cell adhesion and colonization;

Stimulated mineralization potential. 

Moreover, cell morphology changes were influenced by implant surface microstructure. Taken together, these results demonstrate that the minimally invasive MagiCore^®^ could offer good potential for clinical applications through its suitable design and topographical surface properties. 

## Figures and Tables

**Table 1:** Tested implants, their surface treatment, and their composition according to the corresponding titanium grade. Thread dimensions were measured by optical digital microscopy.

IMPLANTS	NOBELPARALLELTM	MAGICORE®
DIMENSION	4.3 × 10 mm 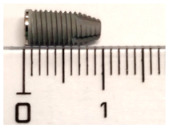	4.5 × 11 mm + 5 mm (neck) 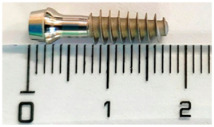
SURFACES TREATMENTS GRADE (COMPOSITION)	*Anodic oxidation*, thick TiO_2_ layer Tubules, curved angles Grade IV (pure titanium): no other alloys, only titanium	*Sandblasting* with calcium phosphate apatitite particles Abrasive: 40 (400 µm)–80 (177 µm) mesh. Sharp angles and summits Grade V (Ti6Al4V): titanium alloy, containing aluminum and vanadium
THREADS DEPTH	0.25 mm	0.9 mm
THREADS WIDTH	0.4 mm	0.25 mm
INTER-THREADS LENGTH	0.6 mm	1.2 mm

**Table 2:** Chemical composition of the tested implant surfaces (chemical elements rate by EDX).

	Ti (%)	Al (%)	V (%)	O (%)	P (%)	C (%)
NOBELPARALLELTM	28.38	-	-	65.24	4.03	2.34
MAGICORE®	47.67	5.30	0.86	19.42	-	26.74

## Data Availability

The data presented in this study are available on request from the corresponding author.

## Supplementary Materials

The following supporting information can be downloaded at: https://www.mdpi.com/article/10.3390/ma15217540/s1, Figure S1: Design and threads morphology of NB™ (left) and MC® (right) by a digital microscope; Figure S2: Morphology of the chemical composition surface (mapping images by EDX). (a) NB™ (b) MC^®^. Scale bars = 1000 µm; Figure S3: Representative images of cell morphology by epifluorescence microscopy at day 3 of exposure to implant extracts: hGF in indirect contact with (a) NB™ and (b) MC®. MG63 in indirect contact with (c) NB™ (d) MC®. Nucleus-blue (DAPI) and cytoskeleton-green (ALEXA Fluor^TM^ 488 Phalloidin), scale bars = 50 µm; Figure S4: Representative images of Alizarin Red S staining of cells mineralized nodules. (a) hGF cells were contacted with NB™ and (b) with MC^®^; (c) MG63 contacted with NB™ and (d) with MC^®^ respectively. Scale bars = 200 µm.
