# Supplementary material for: Comparison of the Biological Behavior and Topographical Surface Assessment of a Minimally Invasive Dental Implant and a Standard Implant: An In Vitro Study
Source: Materials (Basel). 2022 Oct 27;15(21):7540. doi: 10.3390/ma15217540 (PMC9655689; doi:10.3390/ma15217540)
Supplement: Supplementary file 1 [file materials-15-07540-s001.zip › materials-1909795-supplementary.pdf]

materials

Supplementary Material

Comparison of the Biological Behavior and Topographical Surface Assessment of a Minimally Invasive Dental Implant and a Standard Implant: An In Vitro Study

Nina Attik [1,2,*], Marina Phantarasmy [2], Hazem Abouelleil [1,2], Charlène Chevalier [1], Aurore Barraco [1,3], Brigitte Grosgogeat [1,2,3] and Arnaud Lafon [1,2,3]

[1] Laboratoire des Multimatériaux et Interfaces, UMR CNRS 5615, Université Claude Bernard Lyon 1, 69622 Villeurbanne, France
[2] Faculté d'Odontologie, Université Claude Bernard Lyon 1, 69372 Lyon, France
[3] Hospices Civils de Lyon, Service d'Odontologie, 69003 Lyon, France
[*] Correspondence: nina.attik@univ-lyon1.fr

materials

Table of contents:

Figure S1. Design and threads morphology of NB™ (left) and MC® (right) by a digital microscope. **3**

Figure S2. Morphology of the chemical composition surface (mapping images by EDX). (a) NB™ (b) MC®. Scale bars = 1000 µm. **3**

Figure S3. Representative images of cell morphology by epifluorescence microscopy at day 3 of exposure to implant extracts: hGF in indirect contact with (a) NB™ and (b) MC®. MG63 in indirect contact with (c) NB™ (d) MC®. Nucleus-blue (DAPI) and cytoskeleton-green (ALEXA Fluor™ 488 Phalloidin), scale bars = 50µm. **4**

Figure S4. Representative images of Alizarin Red S staining of cells mineralized nodules. (a) hGF cells were contacted with NB™ and (b) with MC® ; (c) MG63 contacted with NB™ and (d) with MC® respectively. Scale bars = 200µm. **5**

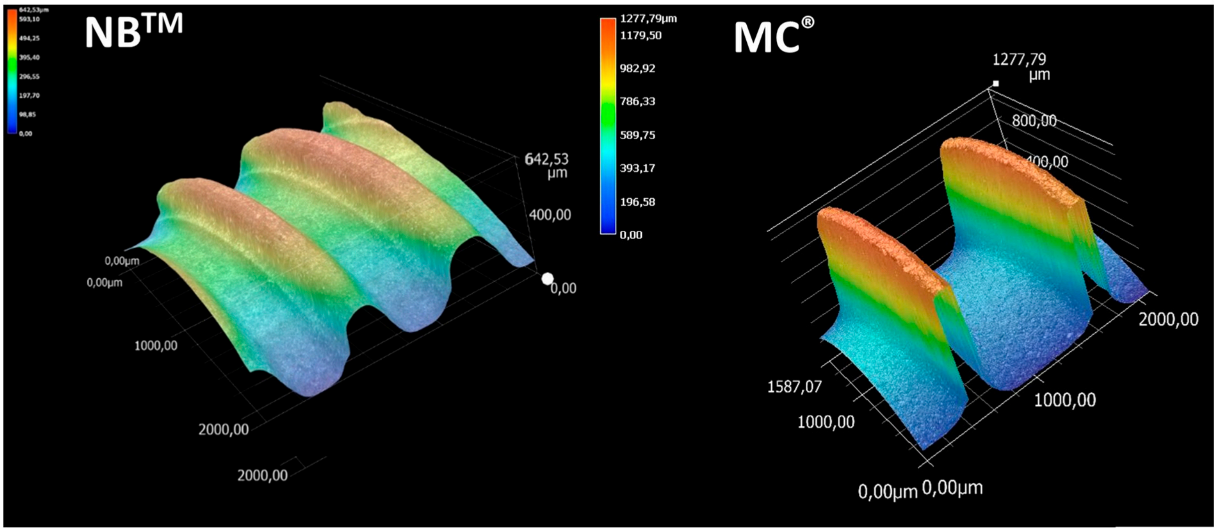

Figure S1. Design and threads morphology of NB™ (left) and MC® (right) by a digital microscope.

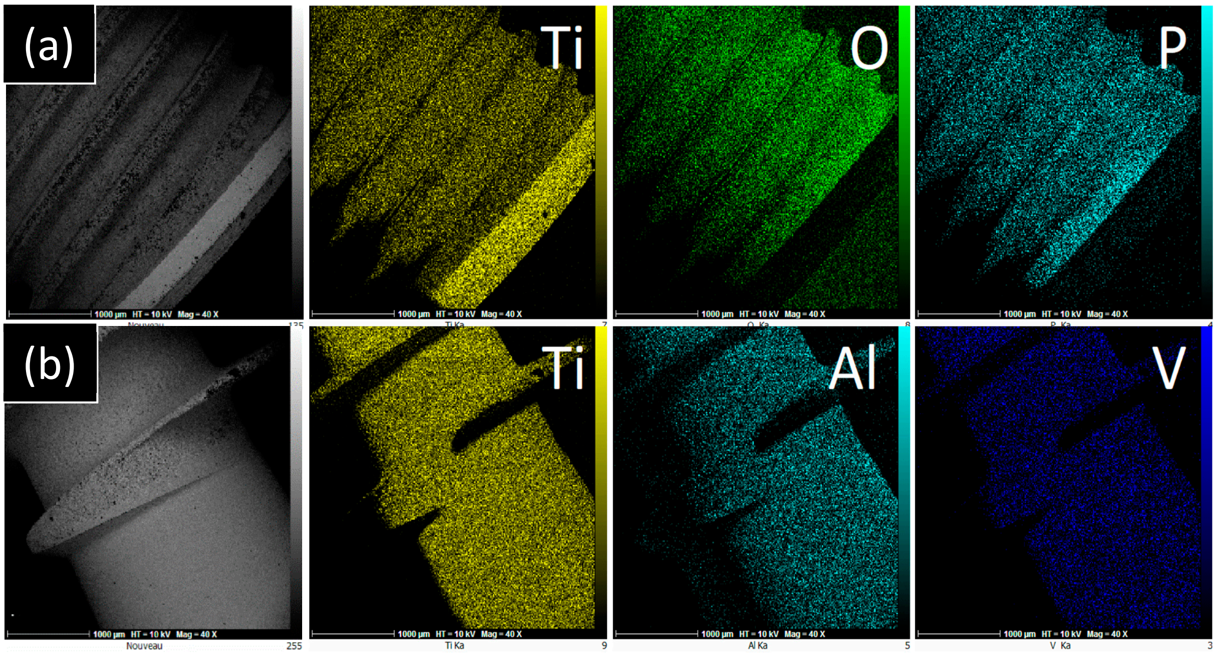

Figure S2. Morphology of the chemical composition surface (mapping images by EDX). (a) NB™ (b) MC®. Scale bars = 1000 µm.

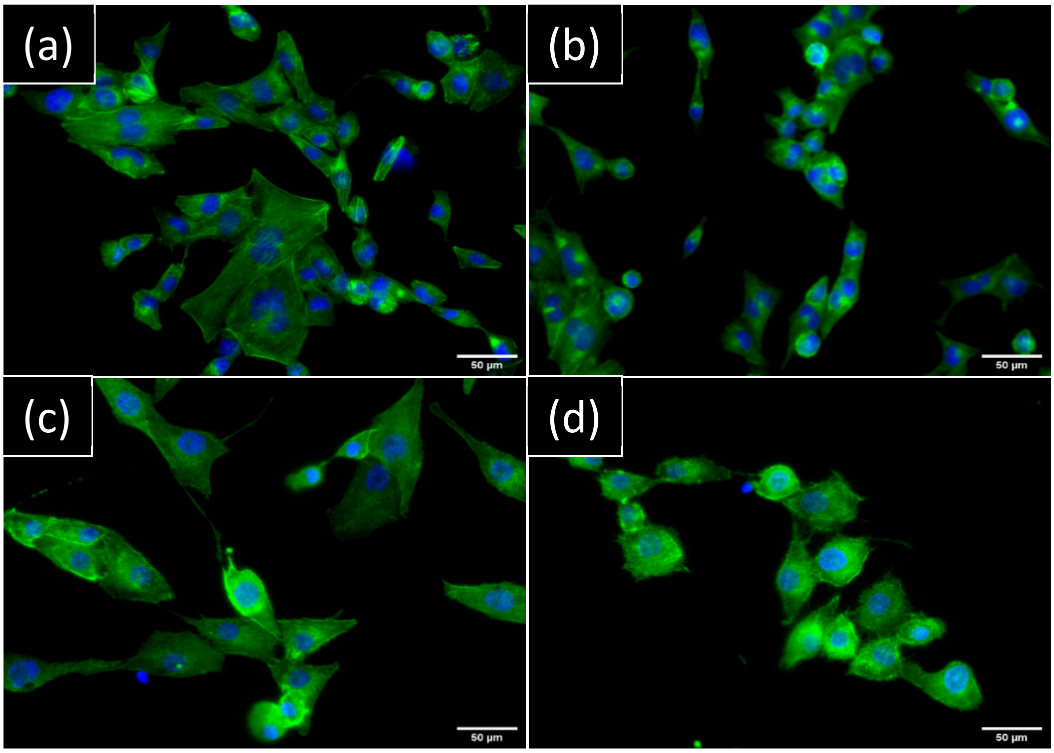

Figure S3. Representative images of cell morphology by epifluorescence microscopy at day 3 of exposure to implant extracts: hGF in indirect contact with (a) NB™ and (b) MC®. MG63 in indirect contact with (c) NB™ (d) MC®. Nucleus-blue (DAPI) and cytoskeleton-green (ALEXA FluorTM 488 Phalloidin), scale bars = 50µm.

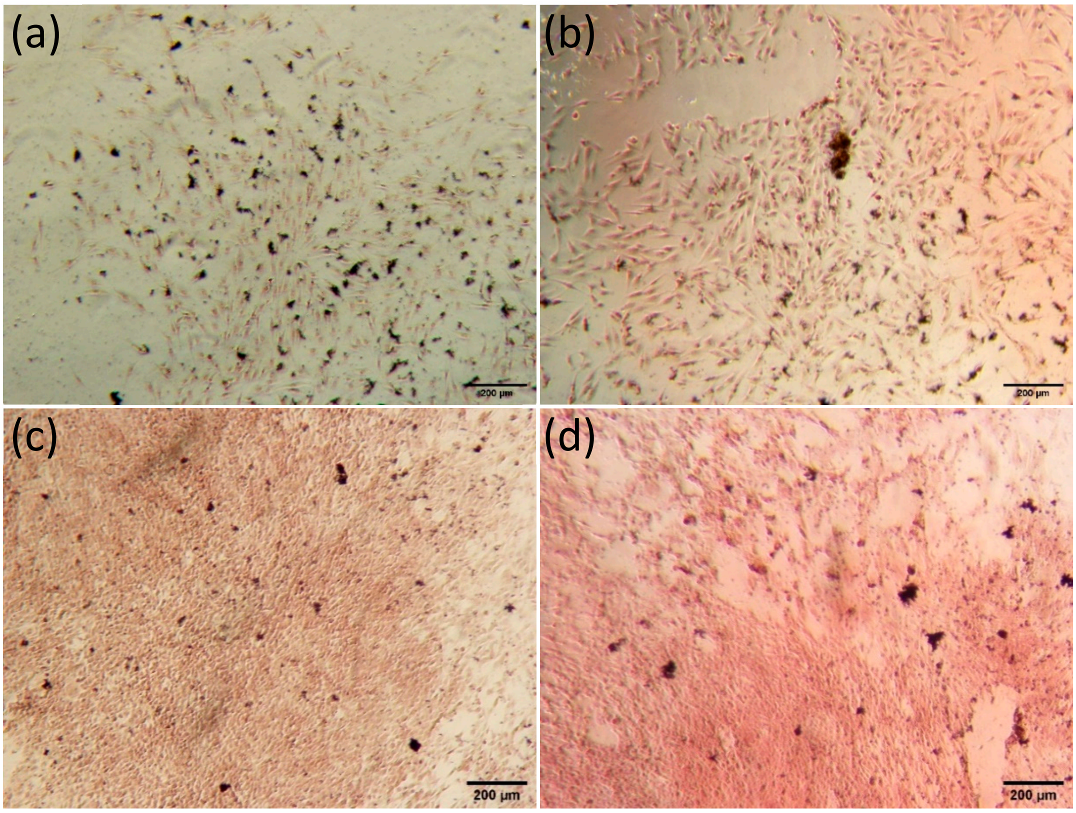

Figure S4. Representative images of Alizarin Red S staining of cells mineralized nodules. (a) hGF cells were contacted with NB™ and (b) with MC® ; (c) MG63 contacted with NB™ and (d) with MC® respectively. Scale bars = 200µm.
